# A Comparative Study of the Management of Anterolateral Instability by Modified Lemaire's Technique Versus Modified Andrews Technique of Lateral Extra-Articular Tenodesis

**DOI:** 10.7759/cureus.83762

**Published:** 2025-05-08

**Authors:** Jerin Jeevo, Sandesh GM, Anoop Pilar, Rajkumar S Amaravathi, Madan Mohan Muniswamy, Bibin Selvin, T A Amith, Megha Ann Mathew

**Affiliations:** 1 Orthopaedics and Traumatology, St. John's Medical College Hospital, Bangalore, IND

**Keywords:** anterior cruciate ligament (acl), arthroscopic acl reconstruction, lateral extra articular tenodesis, modified andrews technique, modified lemaire's technique

## Abstract

Background

Anterior cruciate ligament (ACL) reconstructions together with lateral extra-articular tenodesis (LET), or LET procedures alone, were frequently used to treat ACL tears with anterolateral instability. The choice of the LET procedure is still debated because each technique varies in terms of graft selection and fixation. Therefore, the aim of our study was to compare the functional outcome of the two commonly used lateral extra-articular tenodesis procedures, modified Lemaire and modified Andrews techniques, in combination with ACL reconstruction.

Methods

Fifty patients with ACL tears and anterolateral instability were split into two groups (25 each) for this prospective comparative study. Patients in the first group received treatment using the modified Lemaire technique of lateral extra-articular tenodesis, while patients in the second group received treatment using the modified Andrews technique of lateral extra-articular tenodesis. For both groups, ACL reconstruction was done by the standard transportal technique using a soft tissue graft. Patients of both groups were then evaluated using the Lysholm Knee score at pre-op, six months, and one year post-operative follow-up.

Results

The average age in this study was 31.88 years, consisting of 20 male patients (80%) and five female patients (20%) in the modified Andrews LET group. In the modified Lemaire’s LET group, the average age was 28.92 years, comprising 17 male patients (68%) and 8 female patients (32%). The pre-operative mean Lysholm score for the modified Andrews group was 65.2 +/- 4.72. The post-operative mean Lysholm scores at six months and one year were 86.2 +/- 6.1 and 95.1 +/- 3.58, respectively. The pre-operative mean Lysholm score for the modified Lemaire group was 63.9 +/-2.89. The post-operative mean Lysholm scores at six months and one year were 84.2 +/-7.42 and 94.5 +/- 6.4, respectively. Our study found no statistically significant difference in the Lysholm score between the two groups.

Conclusion

Various LET procedures have been employed to improve ACL reconstruction due to their ability to offer secondary restraint. They also diminish the stress encountered by the intra-articular reconstruction grafts. Consequently, in patients requiring additional safeguarding for the intra-articular graft, including those who are obese, athletes, or possess markedly compromised anterolateral tissues, the incorporation of a LET into an ACL reconstruction may be viable. The selection of the LET procedure, whether modified Lemaire's technique or modified Andrews technique, may be determined at the surgeon's discretion, as both techniques yield comparable functional outcomes according to our study.

## Introduction

The recent "rediscovery" of the anterolateral ligament has focused more on the knee's anterolateral soft-tissue structures [1]. For four decades, the anterolateral structures were considered to be anterior cruciate ligament's (ACL's) secondary stabilizers for regulating the anterolateral rotatory movement [2]. Terry et al. found that 93% of individuals with an ACL tear also had a concurrent anterolateral capsular injury in a clinical trial including 82 consecutive knees [3]. It has been observed that anterolateral rotatory instability is higher in cases of combined ACL and anterolateral structural injury than in cases of isolated ACL injury. Lateral extra-articular tenodesis (LET) procedures, which were previously largely abandoned, are now of interest again due to the realization that the ACL and the anterolateral structures work together in controlling the anterolateral rotatory stability [4]. ACL reconstructions in combination with LET procedures or LET procedures alone were traditionally used to treat ACL tears, which was first suggested as a way to eliminate anterior tibial translation and anterolateral rotatory instability [5]. Various reconstruction techniques have been suggested, used, and later improved to treat anterolateral rotatory instability since the advent of the LET procedure. However, many surgeons were deterred from performing extra-articular reconstructions due to poor long-term outcomes, which included joint over-constraint, residual instability, and graft failure. Some researchers have suggested that the non-anatomical method of these techniques may have led to unfavorable long-term effects, given the observed high failure rate of these procedures [4]. It is difficult to draw unbiased conclusions on the durability and clinical superiority of a particular LET procedure because each technique varies in terms of graft selection and fixation. Therefore, the purpose of this study was to compare the functional outcome between modified Lemaire’s technique and modified Andrews technique, the two commonly used lateral extraarticular tenodesis procedures.

## Materials and methods

This prospective comparative study was carried out between January 2024 and March 2025 at St. John’s Medical College Hospital, Bangalore, India. Patients who were actively involved in professional and recreational sports between the age group of 18 and 45 with ACL tear and anterolateral instability (grade 2 and 3 pivot shift) with adequate preoperative rehabilitation and quadriceps strength (comparable to the opposite side) were included. Patients with extensor lag, infection, multi-ligamentous injuries, revision ACL reconstruction, posterolateral corner injuries, complex meniscal injuries, and lateral compartment arthritis, patients who were not cooperating with the standard physiotherapy protocol, and patients with previous fractures and non-union of proximal tibia, distal femur, and ankle fractures were excluded. A total of 50 patients who met the inclusion criteria were selected and they were randomly assigned to Group A (modified Lemaire’s technique group - 25 patients) and Group B (modified Andrews technique group- 25 patients). Pre-operative Lysholm scores of all patients were calculated at the time of admission. The sample size was calculated using the following formula - $n= 2(Z_{1-\alpha/2} + Z_{1-\beta})^{2}\sigma^{2}/ d^{2}$

where σ: Pooled standard deviation; β: Difference between two group means; Z _1-ß_: Z value for corresponding power; Z _1-α/2_: Two-sided Z value for corresponding α.

Surgical technique

All patients underwent a standard trans-portal ACL reconstruction with a soft tissue graft (hamstring-Semitendinosus and gracilis/ peroneus). In addition, group A patients underwent modified Lemaire’s technique of lateral extra-articular tenodesis, and group B patients underwent the modified Andrews technique of lateral extra-articular tenodesis.

Modified Lemaire’s LET

**Figure 1:**
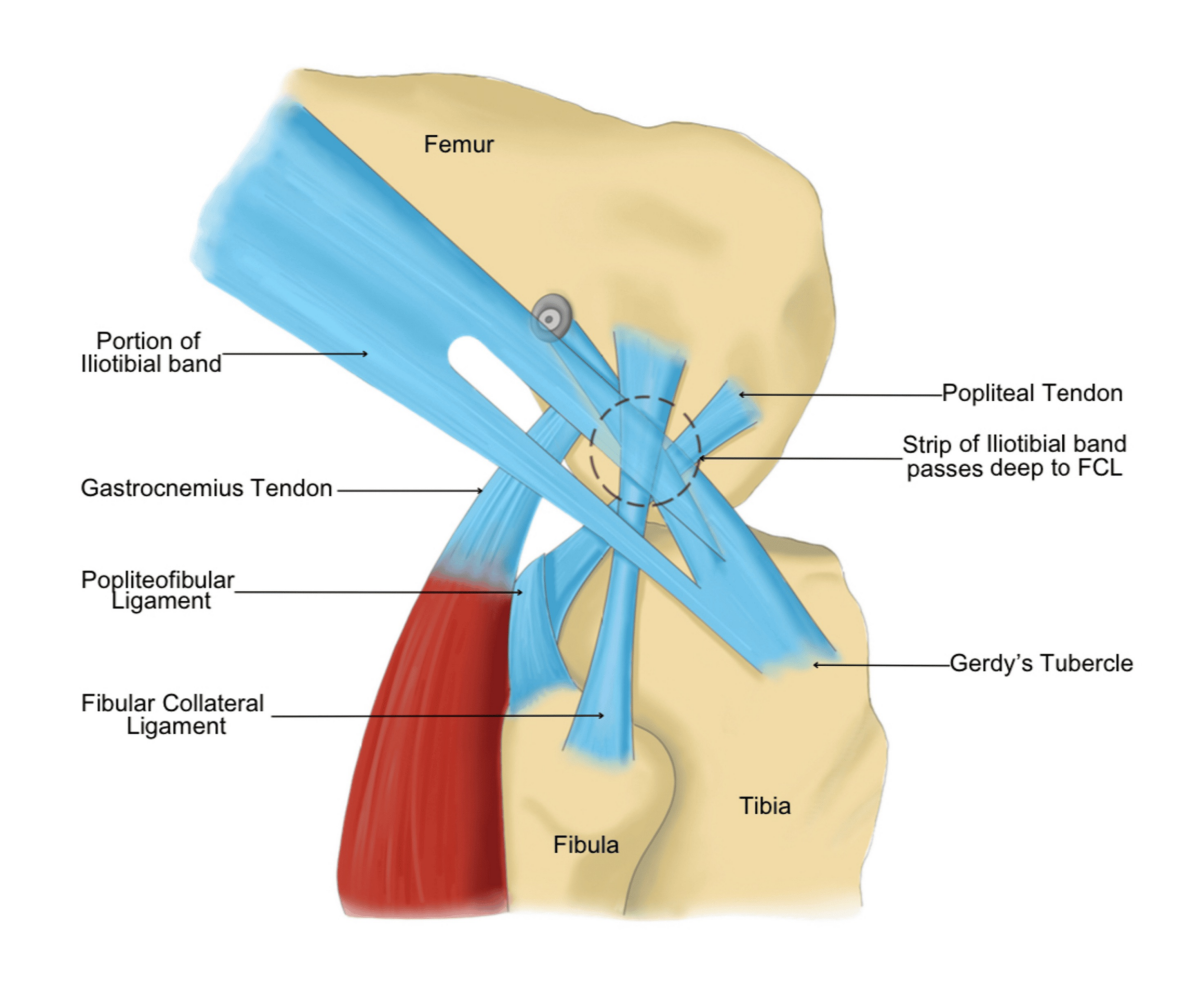
Diagrammatic picture of modified Lemaire’s technique of lateral extra-articular tenodesis The black dotted circle highlights the iliotibial band, which passes deep to the lateral collateral ligament. This is an original image.

**Figure 2:**
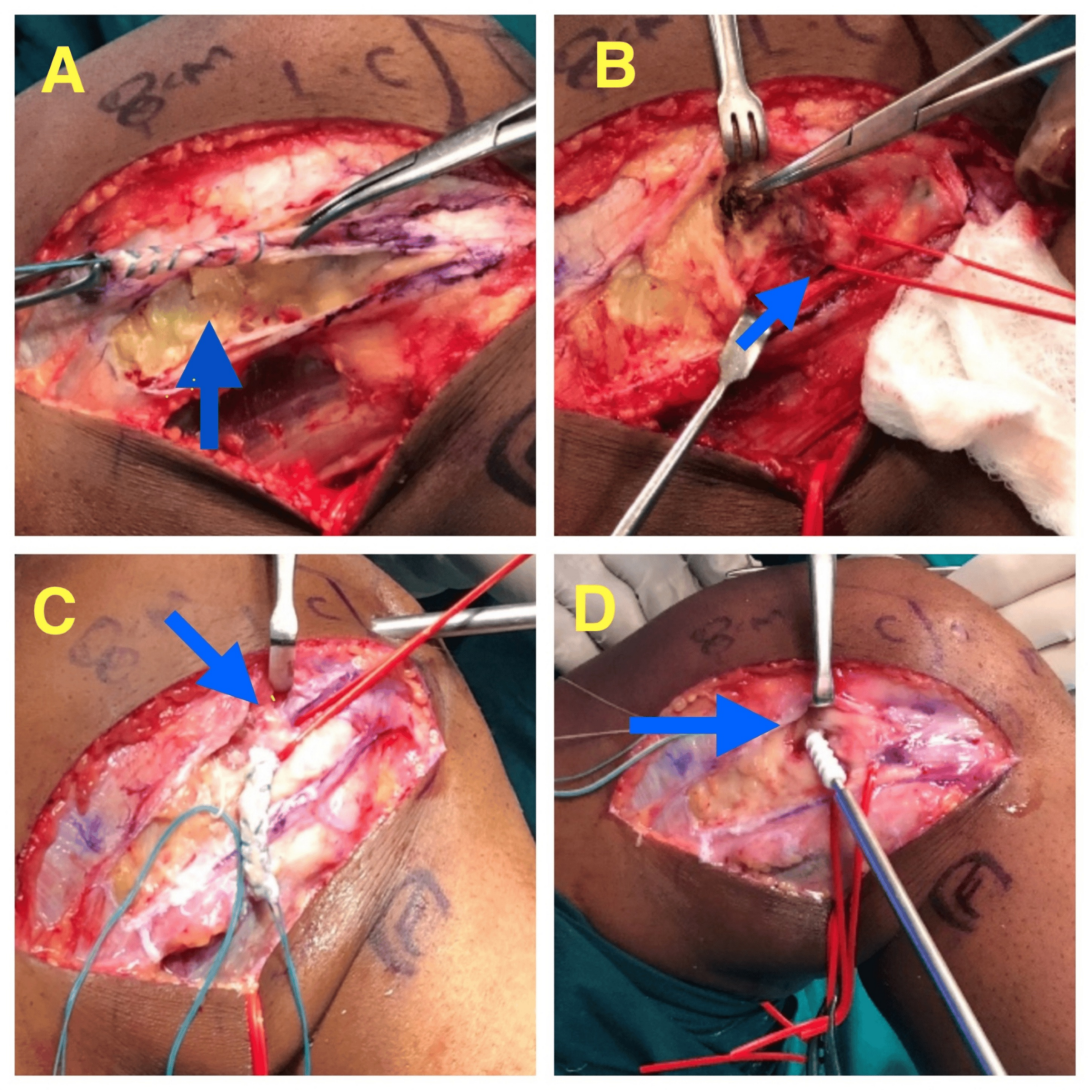
Intra-operative images of modified Lemaire's lateral extra-articular tenodesis A: Blue arrow showing the harvested Iliotibial band graft; B: Blue arrow showing the lateral collateral ligament; C: Blue arrow showing the iliotibial band being passed below the lateral collateral ligament; D: Blue arrowing the iliotibial band being fixed to the F9 point using an interference screw

Patients were positioned supine with the knee at 80 degrees flexion. A lateral skin incision was made and dissection was carried out down to the iliotibial band (ITB). An 8 cm long and 1 cm wide strip of the ITB containing most of the posterior fibers of the deep ITB was harvested, leaving the distal attachment at Gerdy’s tubercle intact. The lateral collateral ligament (LCL) is palpated and then an ITB graft is passed deep to the LCL (Figure 1). With the help of electrocautery, the lateral femoral supracondylar area is cleared and a tunnel is drilled just proximal and posterior to the attachment of LCL with an anteriorly directed trajectory to avoid convergence with the ACL tunnel (Figures 2A-2D). Graft is then passed through the tunnel and fixed with an interference screw with the knee at 60-degree flexion and neutral rotation [6].

Modified Andrews technique

**Figure 3:**
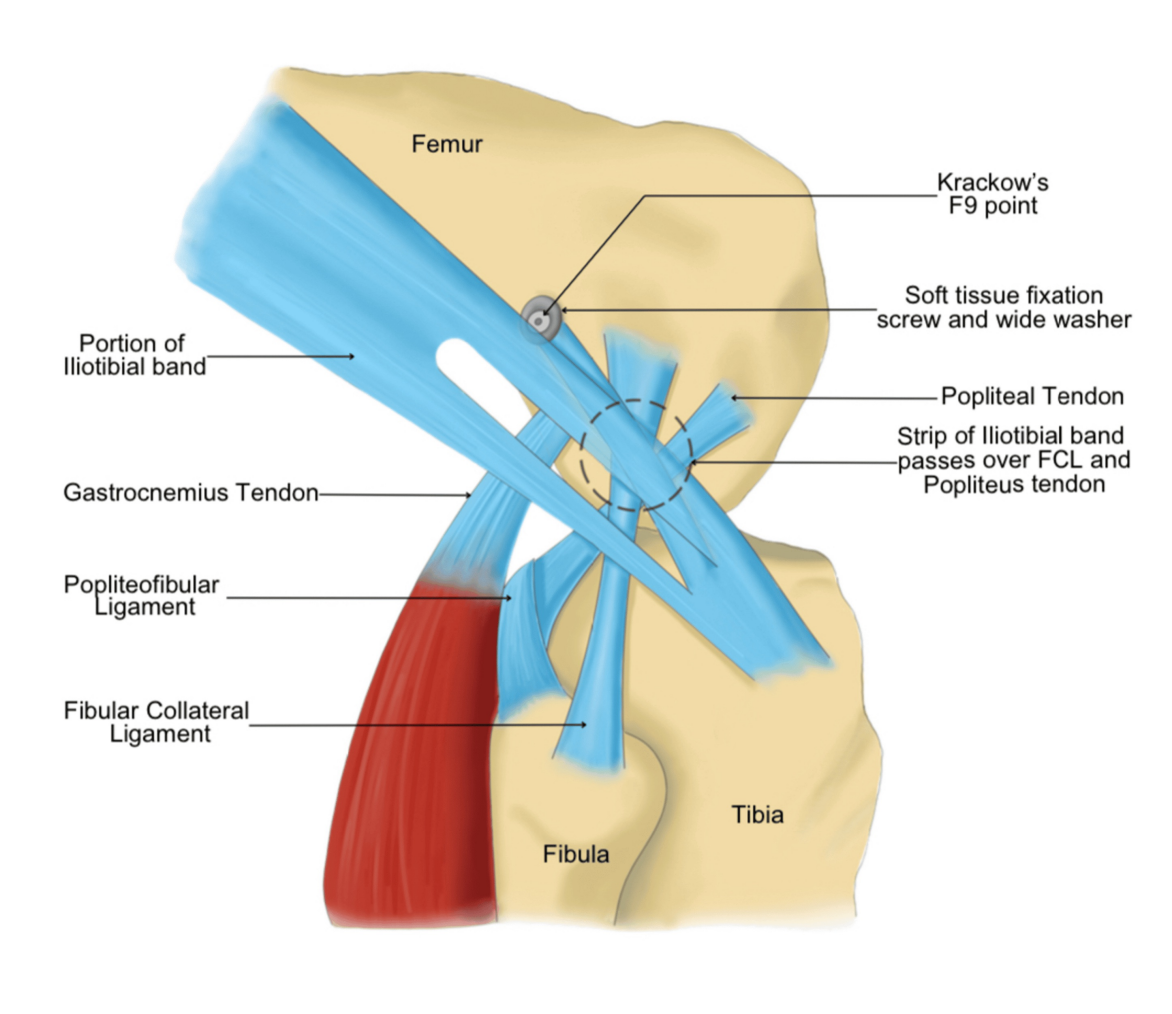
Diagrammatic picture of the modified Andrews technique of lateral extra-articular tenodesis The black dotted circle shows the iliotibial band passing superficial to the lateral collateral ligament. This is an original image.

**Figure 4:**
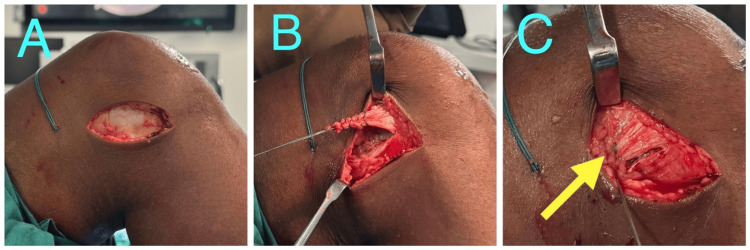
Intra-operative images of modified Andrews technique of lateral extra-articular tenodesis A: Incision; B: Harvested Iliotibial band; C: Yellow arrow showing iliotibial band being taken superficial to the lateral collateral ligament and fixed to the F9 point.

The patient positioning, dissection and ITB graft harvest were done similarly to the modified Lemaire’s technique. The ITB graft is then passed superficial to the lateral collateral ligament (Figure 3). The fixation point (proximal and posterior to LCL) is cleared using electrocautery and a tunnel is drilled with anteriorly directed trajectory to avoid convergence with the ACL tunnel (Figures 4A-4C). The graft is then passed through the tunnel and fixed with an interference screw with the knee in 60 degrees flexion and neutral rotation [7].

Post-operative care and rehabilitation

Following the surgery, patients were started on knee ROM up to 60 degrees and full weight bearing with a long knee brace if there was no meniscal or other intra-articular pathology. Wound was inspected on the second post-operative day and the sutures were removed on the 12th post-operative day. Post-operative rehabilitation was tailored to the patient with the help of a physiotherapist according to the standard protocol.

Follow-up and assessment

Patients were systematically monitored at regular periods following surgery. A single examiner utilized the Tegner-Lysholm knee score to conduct a blinded functional assessment of the knee at six months and one year post-surgery. The score was compared to the pre-operative Tegner-Lysholm score for both groups.

Statistical analysis

Lysholm scores were evaluated at baseline, six months, and 12 months following surgery for patients who underwent either modified Lemaire’s LET or modified Andrews LET. An independent samples t-test was employed to compare scores between the two groups at each time interval. Paired t-tests were utilized to conduct within-group comparisons of Lysholm scores over time (baseline to six months and from six months to 12 months). A p-value of less than 0.05 was considered statistically significant.

## Results

**Figure 5:**
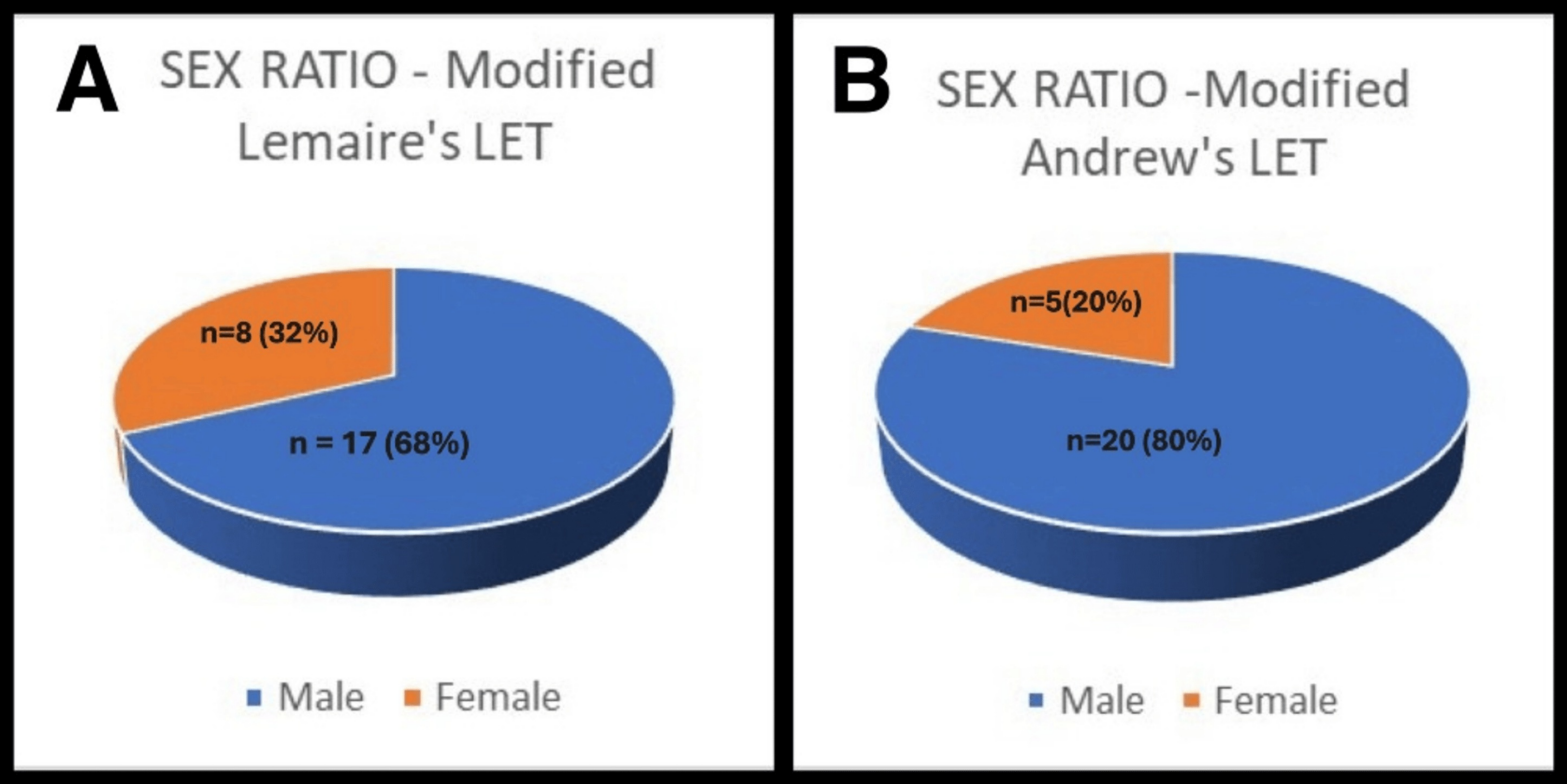
Sex ratio in the two study groups A: Modified Lemaire's LET; B: Modified Andrews LET LET: Lateral extra-articular tenodesis

The mean age group in this study was 31.88 years, comprising 20 male patients (80%) and five female patients (20%) in the modified Andrews technique group. In modified Lemaire’s technique group, the mean age was 28.92 years, with 17 male patients (68%) and eight female patients (32%) (Figures 5A, 5B). 

Among the study participants in modified Andrews LET group, 21 (84%) were recreational athletes and 4 (16%) were professional athletes. In the modified Lemaire's LET group, 19 (76%) were recreational athletes and 6 (24%) were professional athletes (Table 1).

**Table 1:** Demographic factors of the study participants LET: Lateral extra-articular tenodesis

Demographic factors	Modified Andrews LET	Modified Lemaire’s LET
Male patients	20 (80%)	17 (68%)
Female patients	5 (20%)	8 (32%)
Mean age	31.88 years	28.92 years
Nationality	Indian	Indian
Recreational athletes	21 (84%)	19 (76%)
Professional athletes	4 (16%)	6 (24%)

**Figure 6:**
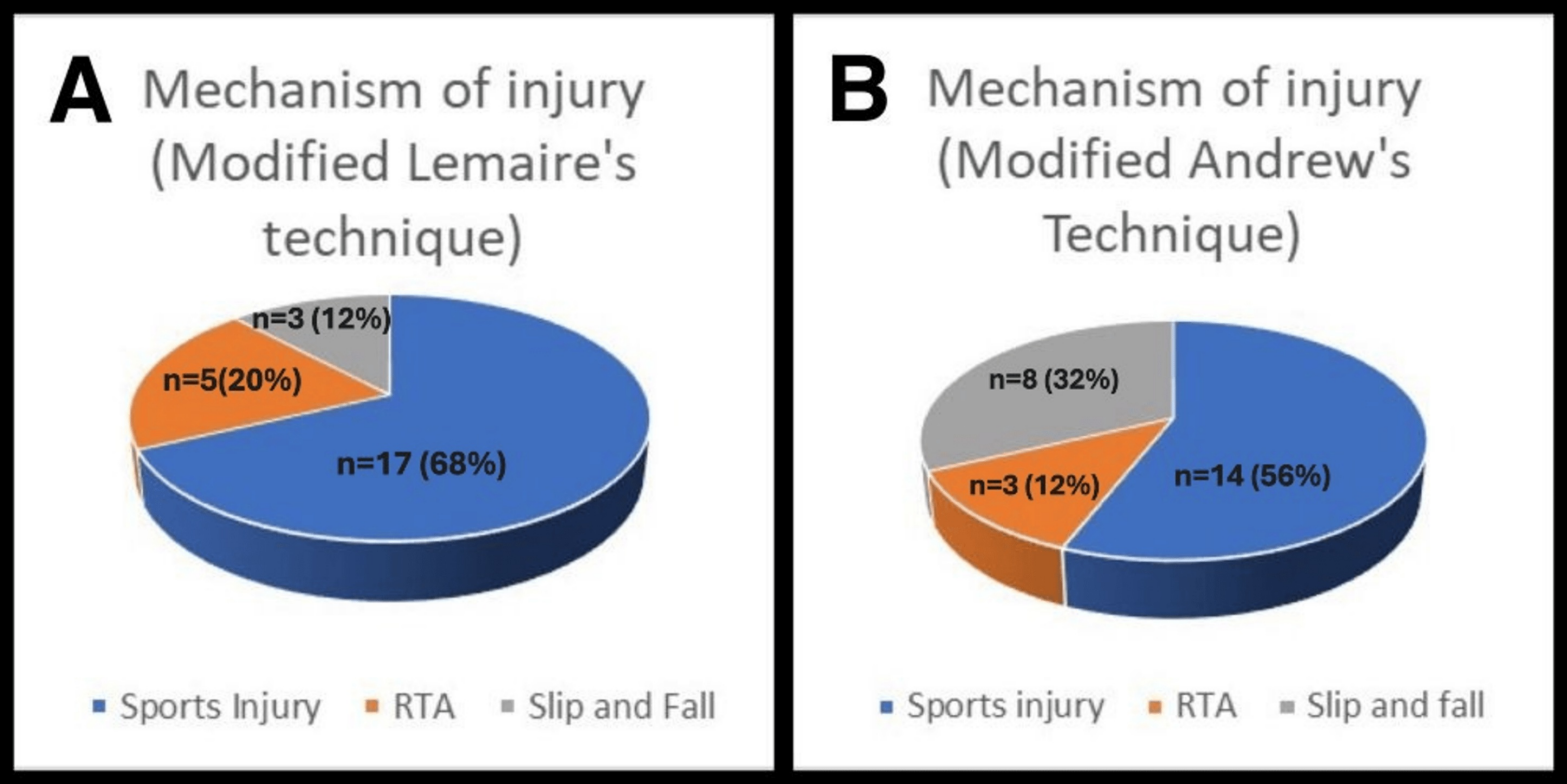
Pie chart showing distribution of various mechanisms of injury in both the study groups A: Modified Lemaire's LET group; B: Modified Andrews LET group LET: Lateral extra-articular tenodesis; RTA: road traffic accident

The primary mechanism of injury was predominantly attributed to sports injuries, with 56% observed in the modified Andrews technique group and 68% in the modified Lemaire’s technique group. Additional factors encompassed road traffic accidents and incidents of tripping and falling (Figures 6A, 6B). 

In modified Andrews's cohort, 64% of patients had a grade 2 pivot shift, while the remaining 36% demonstrated a grade 3 pivot shift. Moreover, 52% of the patients presented with concomitant meniscal injuries, which were addressed during the surgical procedure. In modified Lemaire’s cohort, 56% of patients exhibited grade 2 pivot shift, while the remaining 44% presented with grade 3 pivot shift. Forty-four percent of the patients had concomitant meniscal injuries that were addressed during the surgical procedure. The average duration of the modified Andrews technique was one hour and thirty-three minutes, which was 18 minutes quicker than the modified Lemaire's technique (one hour fifty-one minutes) (Table 2). We hypothesize that this may be attributable to the additional time required to dissect the LCL in the modified Lemaire's technique.

**Table 2:** Table showing the number of patients who had associated meniscal injuries, grades of pivot shift and the operating time in both the groups (modified Andrews and modified Lemaire's) LET: Lateral extra-articular tenodesis

		Modified Andrews LET	Modified Lemaire’s LET
Associated Meniscal injuries		13	11
Pivot shift	Grade 2	16	14
	Grade 3	9	11
Average Operating time		1 hour 33 minutes	1 hour 51 minutes

At baseline, the mean Lysholm score was 63.9 (SD = 2.89) in the modified Lemaire’s LET group and 65.2 (SD = 4.72) in the modified Andrews LET group (Table 3). This difference was not statistically significant (p = 0.253), indicating baseline homogeneity. At six months, the modified Lemaire’s LET group had a mean score of 84.2 (SD = 7.42), while the modified Andrews LET group scored 86.2 (SD = 6.1), with no significant difference (p = 0.323). Similarly, at 12 months, scores were 94.5 (SD = 6.4) for modified Lemaire’s LET and 95.1 (SD = 3.58) for modified Andrews LET (p = 0.700) with no significant difference.

**Table 3:** Comparison of pre-op and post-op Lysholm score of modified Lemaire's LET and modified Andrews LET Statistical analysis was performed using an independent t-test. LET: Lateral extra-articular tenodesis

Lysholm score	Group	Mean	SD	p-value	Test statistic
Pre-op Lysholm score	Modified Lemaire’s LET	63.9	2.89	0.253	-1.16
	Modified Andrews LET	65.2	4.72		
	Group	Mean	SD	p-value	Test statistic
Lysholm score at six months	Modified Lemaire’s LET	84.2	7.42	0.323	-1
	Modified Andrews LET	86.2	6.1		
	Group	Mean	SD	p-value	Test statistic
Lysholm score at 12 months	Modified Lemaire’s LET	94.5	6.4	0.7	-0.382
	Modified Andrews LET	95.1	3.58		

**Figure 7:**
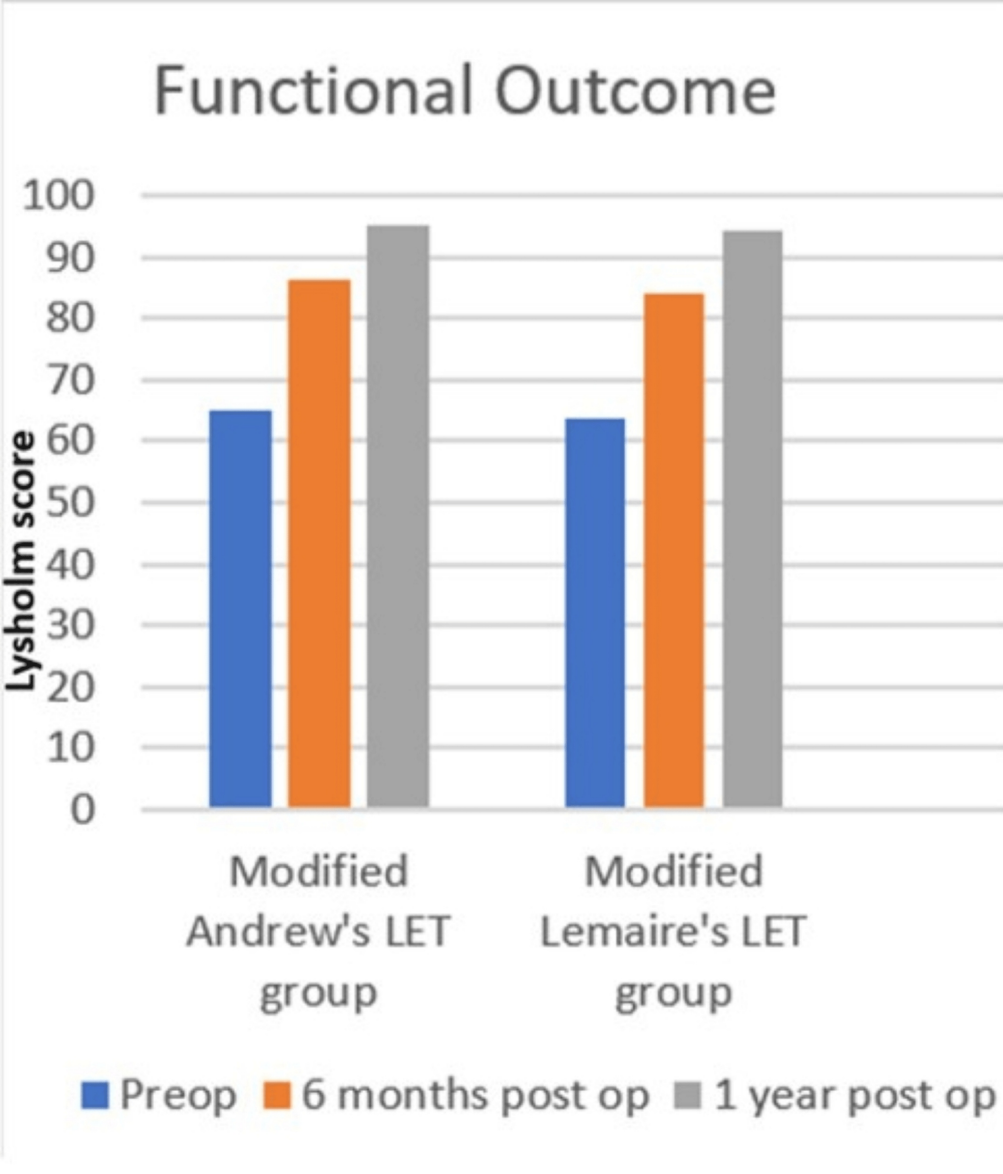
Graph showing pre-op, six months, and 12 months post-operative Lysholm score of modified Andrews and modified Lemaire's LET groups LET: Lateral extra-articular tenodesis

**Figure 8:**
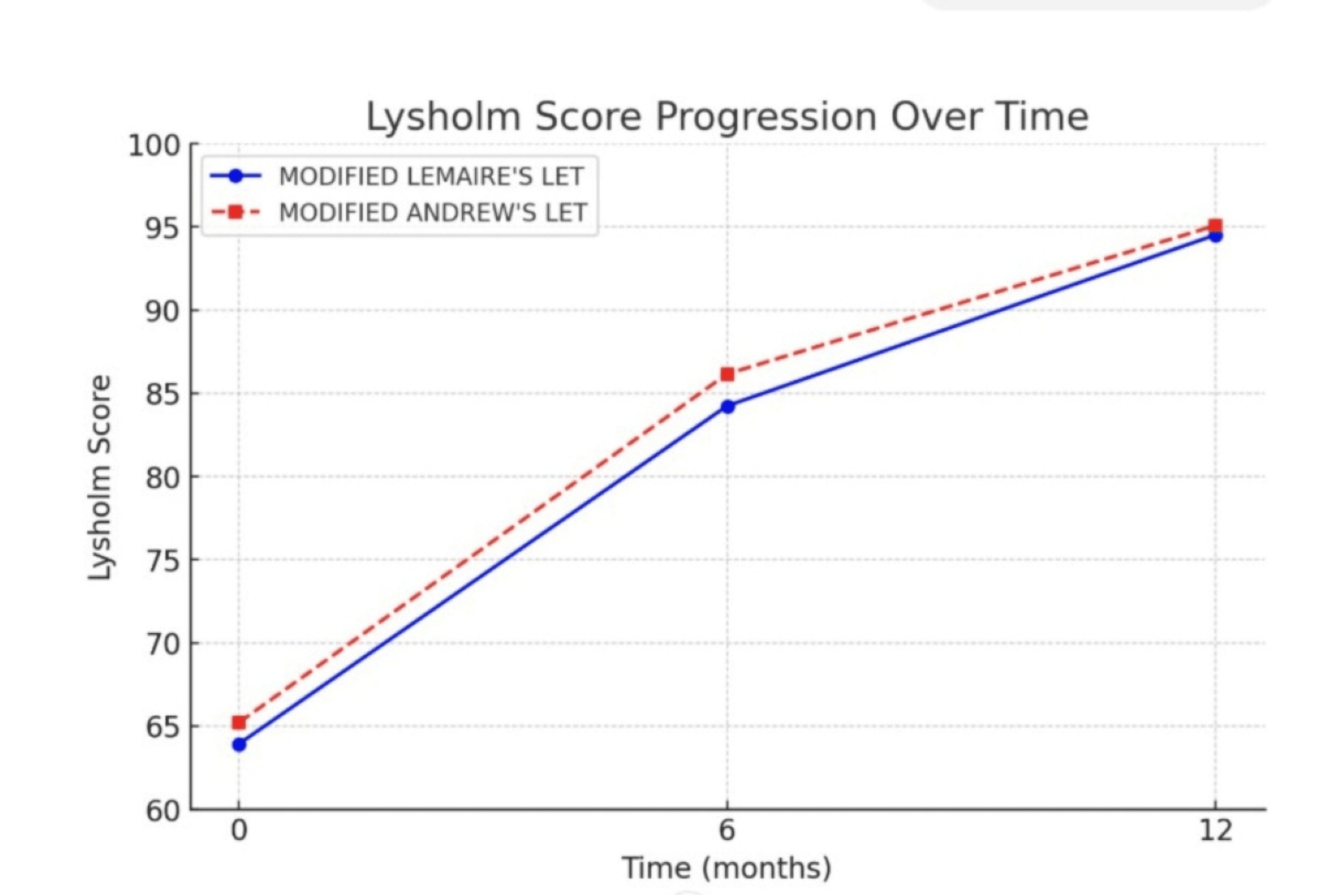
Line chart comparing the Lysholm score of modified Lemaire's LET and modified Andrews LET LET: Lateral extra-articular tenodesis

Both groups showed a steady improvement in Lysholm scores from the preoperative period to 12 months postoperatively. At six months, both techniques demonstrated a marked increase in functional outcomes. By the end of 12 months, both techniques converge closely, reaching nearly equal scores, indicating excellent long-term functional recovery in both procedures, with a minimal difference in outcomes (Figures 7, 8).

Significant improvements in Lysholm scores were observed within each group over time (Table 4). In the modified Lemaire’s group, scores improved from 63.9 at baseline to 84.2 at 6 months (p < 0.0001) and further to 94.5 at 12 months (p < 0.001). Similarly, in the modified Andrews LET group, the score increased from 65.2 at baseline to 86.2 at 6 months (p < 0.0001) and to 95.1 at 12 months (p < 0.0001).

**Table 4:** Table displaying a significant improvement in the Lysholm score of both modified Lemaire's and modified Andrews LET Statistical analysis was performed using a paired t-test. LET: Lateral extra-articular tenodesis

Group	Lysholm score	Mean	SD	p-value	Test statistic
Modified Lemaire’s LET	Pre Lysholm Score	63.9	2.89	<0.0001	-12.765
	Lysholm Score 6m	84.2	7.42		
Modified Andrews LET	Pre Lysholm Score	65.2	4.72	<0.0001	-18.99
	Lysholm Score 6m	86.2	6.1		
Modified Lemaire’s LET	Lysholm Score 6m	84.2	7.42	<0.001	5.25
	Lysholm Score 12m	94.5	6.4		
Modified Andrews LET	Lysholm Score 6m	86.2	6.1	<0.0001	10.059
	Lysholm Score 12m	95.1	3.58		

## Discussion

In order to address residual anterolateral rotatory instability, numerous LET techniques have been put into practice and altered. The majority of studies showed similar biomechanical outcomes despite variations in the surgical techniques, raising earlier doubts over the effectiveness of LET procedures. It was advised in earlier research on LET procedures to fix the graft while keeping the tibia externally rotated. However, non-neutral positioning of the tibia interferes with the "screw home" mechanism because the graft which is anchored externally essentially prevents the tibia from physiologically rotating about its central axis. Furthermore, premature osteoarthritis may occur as a result of abnormal joint kinematics brought on by excessive knee restriction and it has been hypothesized that joint over-constraint causes graft elongation and ultimately graft failure [4]. Therefore in our study, we fixed the graft by keeping the tibia in a neutral position to avoid overconstrain and to allow the normal screw-home mechanism of the knee.

LET surgeries markedly decrease tibial internal rotation to subnormal levels over a spectrum of flexion angles from 0° to 90° in the knees which are ACL deficient. Moreover, LET procedures significantly reduced anterior tibial translation and the stress on the intra-articular graft under an anteriorly directed load; nonetheless, they failed to restore normal anterior stability to the ACL-deficient knee. These results corroborate previous studies indicating that anterolateral rotatory instability in the intact knee is mitigated by the synergistic function of the ACL and anterolateral structures [4].

Kinematic analysis by Neri et al. revealed that LET with the ITB passed over the LCL (superficial LET) exhibits more overconstraint in full flexion than LET with the ITB passed under the LCL (deep LET), indicating the impact of passing the ITB graft over or under the LCL. One theory is that the superficial graft must pass across the epicondyle during flexion, which is a greater distance than a path beneath the LCL, which is nearer the bone. In contrast, a pulley effect is produced, primarily in extension, when the graft is passed beneath the LCL. Consequently, it is likely that the superficial graft will be tighter in flexion and the deep graft would be tighter in extension [8].

Before and after an intra-articular ACL reconstruction, Engebretsen et al. examined the force received by the ACL graft with the addition of a modified Andrews ITB tenodesis. The overall graft force experienced by the ACL graft was significantly reduced by an average of 43% in all four evaluated flexion angles (0°, 30°, 60°, and 90°) with the incorporation of LET into the intra-articular reconstruction. On the other hand, the total graft force dropped by 15% on average across all four tested flexion angles when an ACL reconstruction was added to the LET; however, this decrease was not statistically significant at any of the angles or across all four flexion angles combined [7].

Graft length change patterns varied significantly depending on whether the graft ran deep or superficial to the fibular collateral ligament (FCL), according to Kittl et al. Grafts running deep (medial) to the FCL were inclined to shorten during early knee flexion, while those running over (lateral to) the FCL tended to increase the length. According to Kittl et al., grafts that extended deep into the FCL exhibited "desirable" length change patterns, meaning that throughout knee flexion and extension, their length was largely constant [9].

Kittl et al. also found that any combination of tibiofemoral reconstructions that coursed deep to the FCL and placed proximal to the lateral epicondyle was almost isometric between 0° and 90°. There was only a little increase in length on knee extension, suggesting that the lateral tibial plateau's anterior subluxation may be prevented [9].

According to a study by Krackow et al., the graft attachment points T-3 (Gerdy's tubercle) and F-9 (proximal and posterior to the lateral epicondyle) are considered to have optimal characteristics for addressing the pivot shift concern. Observations indicate that a ligament reconstruction situated at these two points of attachment is most effective between 15 and 35° of flexion, the position where the Lachman sign is positive and the pivot shift occurs [10].

A study performed by Xu et al. compared the biomechanical properties of 3 LET procedures: deep LET-IT (intra-tunnel fixation), superficial LET-IT (intra-tunnel fixation), and deep LET-C (cortical fixation). In this study, the three examined LET procedures diminished anterior, internal rotational, and anterolateral laxities in anterolateral deficient knees, reestablishing these factors to the original baseline of the normal state across the majority of the flexion angles. All LETs over-constrained in deep flexion, but the deep LET-C had less over-constraint in anterior translation and internal rotation than the superficial and deep LET-IT states [11].

According to a study by Aryana et al., the incorporation of the LET procedure with ACL reconstruction yields improved functional outcomes as indicated by the IKDC score and the incidence of graft failure was observed to be reduced in the ACLR plus LET cohort [12]. Also according to a meta-analysis by Agarwal et al., LET in conjunction with ACLR yields enhanced mechanical outcomes and reduced incidence of ACL re-ruptures [13].

Between modified Lemaire’s (deep LET) technique and modified Andrews (superficial LET) technique, there was no significant difference in the post-operative Lysholm score at six months and one year follow-up according to our study. The operating time for modified Andrews technique was 18 minutes faster than the modified Lemaire’s technique which we suggest this may be due to the extra time needed to dissect the LCL in the modified Lemaire technique. Even though most of the literature indicates that modified Lemaire's LET has superior biomechanical properties, in our study, there was no significant difference in the clinical outcome, as measured by the Lysholm score, between modified Andrews LET and modified Lemaire's LET.

Limitations of the study

The one-year follow-up duration inadequately reflects long-term outcomes, including potential re-rupture or graft failure, necessitating extended research to validate the durability of these results. A further limitation is the small sample size of our study. Subsequent research should investigate the biomechanical characteristics of lateral extra-articular tenodesis more comprehensively, directly comparing it with alternative grafts in bigger, randomized controlled trials.

## Conclusions

LET procedures have been employed to augment ACL reconstruction due to their capacity to offer secondary restraint and to diminish stress experienced by intra-articular reconstruction grafts. Therefore, in patients who may need extra protection for the intra-articular graft, such as those who are obese, athletes, or who have significantly impaired anterolateral tissues, adding a LET to an ACL reconstruction may be useful. The choice of LET procedure between modified Lemaire's technique and modified Andrews technique can be left to the surgeon's discretion since both these techniques provide a similar functional outcome according to our study.
